# The ‘Conceptual Distance Effect’ in the Causal Effects Under Experimental Manipulation Between Attitude and Stereotype

**DOI:** 10.3390/bs16020287

**Published:** 2026-02-17

**Authors:** Yang Yang, Xue Bai, Jiejie Liao, Yujie Chen, Lei Mo

**Affiliations:** 1 Department of Experimental Psychology, Ghent University, 9000 Ghent, Belgium; 2 Shenzhen Zhili School, Shenzhen 518000, China; 3 School of Education, Guangdong University of Education, Guangzhou 510303, China; 4 Center for Studies of Psychological Application, South China Normal University, Guangzhou 510631, China; 5 Key Laboratory of Brain, Cognition and Education Sciences, Ministry of Education, South China Normal University, Guangzhou 510631, China

**Keywords:** attitude, stereotype, belief, causal effects under experimental manipulation, conceptual distance, evaluative conditioning, social cognition

## Abstract

Understanding how attitudes and stereotypes influence each other is central to social cognition, yet prior findings have been inconsistent, with some indicating strong connections and others suggesting separation. To help explain these discrepancies, we introduce the construct of conceptual distance, defined as the evaluative proximity between attitude objects and stereotypical trait dimensions (e.g., warmth, morality, competence). Across four experiments, we first measured conceptual distance using a forced-choice task that estimated how closely each trait dimension aligns with positive or negative valence. We then tested whether the strength of causal effects between attitudes and stereotypes corresponds to these distances. Attitudes or stereotypes were manipulated using evaluative conditioning (EC), and their effects were measured through either explicit self-report ratings or Implicit Association Tests (IATs). Results consistently showed stronger causal effects for stereotype dimensions that were evaluatively closer to attitudes (warmth and morality) than for more distant ones (competence). These findings offer initial evidence for a correspondence between conceptual distance and the strength of experimentally induced influence. The study contributes to theories of causal cognition and social representation, and offers implications for designing interventions that aim to reduce stereotype-based bias and promote more flexible social inferences.

## 1. Introduction

Attitudes are generalized valence attributions to other individuals or groups (e.g., white-good/black-bad), whereas stereotypes are beliefs about specific trait attributes commonly associated with social groups (Cuddy et al., 2009; Yzerbyt, 2016). These trait-based beliefs can be either positive or negative, such as associating some groups with warmth and intelligence, and others with laziness or dishonesty (Fiske et al., 2002; Cuddy et al., 2009; Bai et al., 2020). Attitudes and stereotypes are both important constructs in social cognition. Understanding the relationship between attitude and stereotype is one of the hot issues in the field of social cognition. Previous studies have employed both explicit and implicit measures to investigate the attitude–stereotype relationship. Explicit studies of the correlational relationship have shown that attitude and stereotype are consistent. For example, Brigham found increased liking of African Americans predicted increased endorsement of positive traits, such as confidence, and decreased endorsement of negative traits, such as laziness (Brigham, 1972). The high correlation may be due to the evaluative nature of most stereotype concepts, such as the finding that Black people are described as violent and lazy (Ghavami & Peplau, 2013; De Leon & Rosette, 2022).

Consistent with these findings, many studies have found a high correlation between implicit attitudes and implicit stereotypes (Kurdi et al., 2019; Rubinstein et al., 2023). However, other studies have shown that attitudes and stereotypes are not always related. For example, Ho and Jackson (2001) found that White Americans hold ambivalent attitudes toward Asian Americans, simultaneously endorsing positive stereotypes such as intelligence while also maintaining negative evaluations. In addition, people have positive attitudes toward older people but also believe that older people have negative traits such as cognitive and physical decline (Cuddy & Fiske, 2002; Lin et al., 2005; Shakeri & North, 2025). This is probably because self-reported group beliefs are often based on consensual cultural stereotypes (Kurdi et al., 2019). As for implicit studies, Amodio and Devine (2006) found no relationship between attitudes and stereotypes of Black Americans. They measured implicit stereotypes related to mental and physical traits. The reason for this null result may be that the stereotype materials used in this study were neutral, and the evaluative implications of stereotype content may influence the pattern of attitude–stereotype relationships.

Although most correlation studies have found significant relationships between attitude and stereotype, many others have found no relationship. Moreover, the limited number of studies on causal effects under experimental manipulations has yielded mixed results. Gawronski et al. (2008) found that training participants to affirm anti-stereotype content significantly reduced their negative perceptions of the group, suggesting that changes in stereotypes can lead to changes in implicit attitudes. Kurdi et al. (2019) found that experimentally induced changes in attitudes toward a fictional group led to changes in participants’ perception of the group’s traits (stereotypes). Additionally, Phills et al. (2020) examined the causal effects under experimental manipulation between implicit attitudes and stereotypes by manipulating the overall valence of trait attributions. They found that participants’ evaluations of the group varied accordingly. However, their study examined only the overall valence of stereotypes. In practice, group stereotypes are multi-attribute representations, composed of several specific traits that carry distinct semantic and behavioral implications. Therefore, it is necessary to investigate how attitudes causally influence individual stereotype components, rather than treating stereotypes as an undifferentiated evaluative whole.

The stereotype content model (SCM) classifies social cognitive content into two primary dimensions: warmth and competence (Fiske et al., 2002; Luna Cortes, 2024). SCM suggests that beliefs about specific traits (stereotypes) can become dissociated from general evaluations (attitudes). Crandall et al. (2011) examined whether stereotypes emerge from pure attitudes and found that attitude shifts via evaluative conditioning changed warmth-related stereotypes but not competence-related ones. This pattern was explained by the “warmth-first” perspective where individuals prioritize warmth in evaluation and response (e.g., Eisenbruch & Krasnow, 2022). However, this explanation only accounts for the warmth dimension and fails to explain the absence of an effect on competence. To account for inconsistent findings across studies, we argue that a new explanatory mechanism is needed.

While previous work (e.g., Esses et al., 1993) has emphasized the association strength between stereotypes and specific social groups as a moderator of intergroup evaluations, our approach is conceptually distinct. We propose that the evaluative closeness between trait concepts (e.g., “honest,” “lazy”) and evaluative meanings (e.g., “good,” “bad”)—which we refer to as conceptual distance—is a key factor shaping the causal links between stereotypes and attitudes. Unlike stereotype–group linkage strength, which concerns how strongly a trait is tied to a particular group, conceptual distance reflects how closely a trait is psychologically aligned with evaluative valence. This distinction enables us to examine variation in the potential causal influence of different stereotypes, regardless of their group associations.

Our view is inspired by the spreading activation model (Collins & Loftus, 1975), which suggests that concepts are organized based on semantic distance, with closer concepts more strongly connected. Both attitudes and stereotypes can be represented by multiple conceptual nodes (e.g., “pleasant” for attitude; “friendly” for stereotype). Building on this idea, we propose a conceptual distance effect: the closer a stereotype trait is to evaluative meaning, the more likely it is to exert a stronger causal effect on attitudes—and vice versa. Prior studies have not systematically examined the relationship between attitudes and stereotypes from this evaluative proximity perspective.

Our work addresses this gap by testing the conceptual distance effect across three key stereotype dimensions—warmth, morality, and competence—and in both causal directions, using both explicit and implicit measurement approaches. While warmth and competence are the core dimensions in the SCM, morality has often been treated as a subcomponent of warmth. However, accumulating research highlights morality as a theoretically distinct and evaluatively central dimension. Recent studies have shown that morality-related traits (e.g., honesty, fairness) play a uniquely powerful role in impression formation and social evaluation—sometimes even surpassing warmth in importance (e.g., Brambilla et al., 2021; Kunst et al., 2025; Luttrell et al., 2022). Based on this growing body of work, we treat morality as a separate dimension in our design, alongside warmth and competence. Together with prior evidence for the “warmth-first” principle, we hypothesized that warmth and morality are conceptually closer to evaluative meaning than competence. Building on this assumption, we further predicted that stereotype dimensions with greater conceptual proximity—specifically warmth and morality—would exhibit stronger bidirectional causal effects under experimental manipulations with attitudes.

To comprehensively examine this hypothesis, we designed a series of studies combining both explicit and implicit approaches. Across all experiments, evaluative conditioning was used as the core paradigm to manipulate either attitudes or stereotypes, following well-established protocols (e.g., Phills et al., 2020; Moran et al., 2025). As a first step, a pre-experiment was conducted to test whether warmth and morality are conceptually closer to attitudes than competence, which set the foundation for subsequent investigations. Experiments 1, 2a, 2b, and 2c examined the bidirectional causal effects under experimental manipulation between stereotypes and attitudes using explicit measures. Specifically, Experiment 1 explored how attitudes affected stereotypes across dimensions, while Experiments 2a–2c investigated how stereotypes of different dimensions influenced explicit attitudes. Experiments 3, 4a, 4b, and 4c mirrored this structure using implicit measures on dependent variables: Experiment 3 focused on how conditioned attitudes influenced implicit stereotypes, and Experiments 4a–4c explored how stereotypes affected implicit attitudes.

## 2. Pre-Experiment

This experiment aimed to examine the “conceptual distance” between evaluative attitudes and three core stereotype dimensions—warmth, morality, and competence—by measuring trait selection frequencies under positive and negative valence conditions.

### 2.1. Participants

Participants were undergraduate students recruited from South China Normal University. All participants had normal or corrected-to-normal vision and provided informed consent prior to participation. In the pre-experiment, 71 participants (22.36 ± 1.98 years; 51 females) completed a series of forced-choice tasks assessing trait selections across dimensions.

### 2.2. Materials

#### 2.2.1. Trait Words

A total of 180 trait words (60 per dimension: warmth, competence, morality) were compiled from previous literature (Brambilla et al., 2011; Fiske et al., 2007, 2002; Leach et al., 2007). Words were rated on representativeness using a 5-point scale.

The ratings were conducted independently by three graduate students in the field of social psychology from South China Normal University. Based on the averaged ratings, the top 90 words (30 per dimension; 15 positive, 15 negative) were selected for use in the experiments (see Table S1 in the Supplementary Materials).

#### 2.2.2. Stimuli Images

Valenced pictures used for evaluative conditioning were selected from the International Affective Picture System (IAPS) (Lang et al., 2008).

#### 2.2.3. Questionnaires and Scales

Attitude Questionnaire. The questionnaire consisted of 21 items (e.g., “I like to make friends with people from Planet X”), developed based on the cognition–affect–behavior structure (McCoach et al., 2013). All items were drawn from established attitude questionnaires used in previous studies, with wording adapted to the context of the present research (Crites et al., 1994; Ho & Jackson, 2001; Kenny et al., 2018) (see Table S2 in the Supplementary Materials).

Continuous Trait Scale. A 5-point Likert scale was used to measure explicit stereotypes.

Implicit Association Test (IAT). Implicit attitudes and stereotypes were assessed following standard IAT procedures (Greenwald et al., 1998).

All questionnaires were administered in Chinese. The items were based on original versions reported in prior literature, with only minor contextual adjustments made to fit the experimental setting.

### 2.3. Experimental Design and Procedures

**Figure 1:**
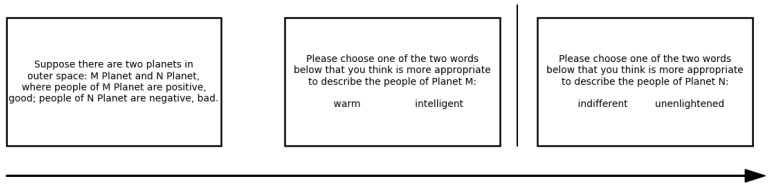
Procedure of word forced-choice task.

Two within-subject tasks were implemented, each under a different valence condition. In the positive valence condition, participants selected trait words that best described fictional group members associated with positive evaluation. In the negative valence condition, the task was repeated using groups associated with negative evaluation. In both cases, stereotype dimension (warmth, morality, competence) was manipulated within subjects, and trait selection frequency was used to estimate perceived evaluative proximity. Participants selected the trait word most descriptive of fictional group members (e.g., Planet M vs. Planet N) in a series of forced-choice trials (see Figure 1 for the details of the procedure). Selection frequency served as a proxy for how strongly each stereotype dimension was mentally linked to evaluative meaning (“good” or “bad”). We term this inferred psychological closeness “conceptual distance”.

### 2.4. Results

For the pre-experiment, each forced-choice response was treated as a trial-level observation. The dependent variable was the selected trait dimension (warmth, morality, competence). We estimated multinomial logistic regression models separately for positive and negative conditions, with competence as the reference category.

**Figure 2:**
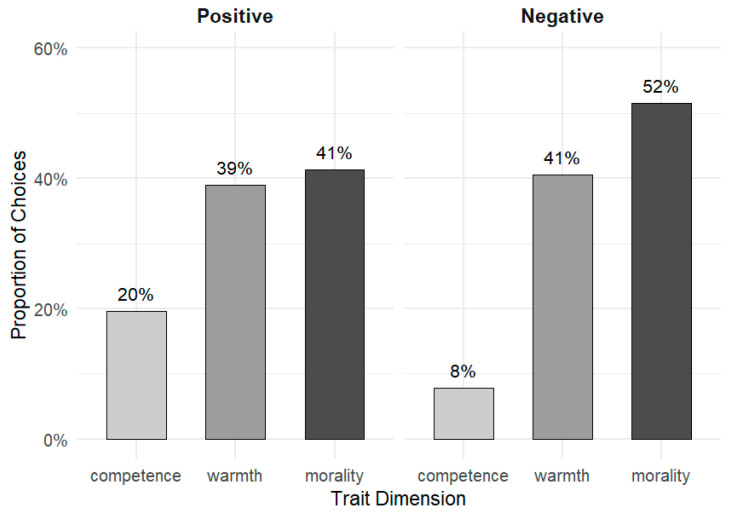
Proportion of words selected from each trait dimension in the pre-experiment, separately for positive and negative conditions. Across both conditions, participants were significantly more likely to select morality-related and warmth-related words than competence-related words, supporting the conceptual distance hypothesis.

In the positive condition, participants were significantly more likely to select morality-related words (b = 0.75, SE = 0.10, OR = 2.11, *p* < 0.001) and warmth-related words (b = 0.69, SE = 0.10, OR = 1.99, *p* < 0.001) than competence-related words. In the negative condition, this pattern was even more pronounced: participants were much more likely to select morality-related words (b = 1.88, SE = 0.08, OR = 6.52, *p* < 0.001) and warmth-related words (b = 1.64, SE = 0.08, OR = 5.14, *p* < 0.001) over competence-related words (see Figure 2).

These results provide robust evidence for the conceptual distance hypothesis: across both positive and negative contexts, morality and warmth consistently appear more closely associated with attitudes than competence.

## 3. Experiment 1

Experiment 1 aimed to test whether experimentally induced evaluative attitudes could causally influence explicit stereotype judgments across the dimensions of warmth, morality, and competence.

### 3.1. Participants

56 participants (21.24 ± 2.03 years; 44 females) were recruited from South China Normal University. All participants had normal or corrected-to-normal vision and provided informed consent prior to participation.

### 3.2. Experimental Design and Procedures

This study employed a within-subject design. The independent variable was attitude valence (positive vs. negative), manipulated through evaluative conditioning. The dependent variables were explicit stereotype ratings across three dimensions (warmth, morality, competence). This experiment consisted of two phases: a learning phase and a test phase.

#### 3.2.1. Learning Phase

To manipulate attitudes, we employed an evaluative conditioning (EC) paradigm. Two fictional social groups were constructed: Planet X (with surname “Xiao”) and Planet L (with surname “Lu”). Participants viewed repeated pairings of group names with emotionally valenced images. Specifically, names from Planet X were consistently paired with positive images, while names from Planet L were paired with negative images. These images were selected from the International Affective Picture System (IAPS; Lang et al., 2008) and were unrelated to stereotype-relevant content.

The learning phase consisted of six blocks. After every two blocks, participants were asked manipulation check questions such as “What do you think of people from Planet X?” and “What do you think of people from Planet L?” to assess the effectiveness of the conditioning process.

#### 3.2.2. Test Phase

Following the learning phase, participants completed stereotype assessments using a 5-point continuous trait rating scale. They were asked to rate their general impressions of each group (e.g., “I think people from Planet X are friendly”), with response options ranging from 1 = Strongly disagree to 5 = Strongly agree. The trait words used for the ratings were selected from a validated vocabulary list constructed in a pre-experiment, covering key stereotype dimensions: warmth, morality, and competence. This design enabled a controlled examination of how experimentally induced attitudes influence explicit stereotype judgment.

### 3.3. Results

A 2 (Valence of attitude: Positive vs. Negative) × 3 (Stereotype Dimension: Warmth, Morality, Competence) repeated-measures ANOVA was conducted on explicit stereotype ratings. The analysis revealed a significant Valence × Dimension interaction, F(2, 106) = 118.56, *p* < 0.001, η^2^ = 0.24, indicating that the impact of evaluative attitudes on stereotype judgments varied across dimensions.

Follow-up paired-sample *t*-tests showed that participants rated the target group significantly warmer when they held a positive attitude (M = 4.15, SD = 0.47) compared to a negative attitude (M = 2.50, SD = 0.68), t(53) = 12.06, *p* < 0.001, d = 1.64. Similarly, morality ratings were higher in the positive condition (M = 3.88, SD = 0.50) than in the negative condition (M = 2.79, SD = 0.77), t(53) = 6.93, *p* < 0.001, d = 0.94. In contrast, competence ratings did not significantly differ between the positive (M = 3.44, SD = 0.55) and negative (M = 3.45, SD = 0.63) conditions, t(53) = −0.06, *p* = 0.95, d = −0.01.

**Figure 3:**
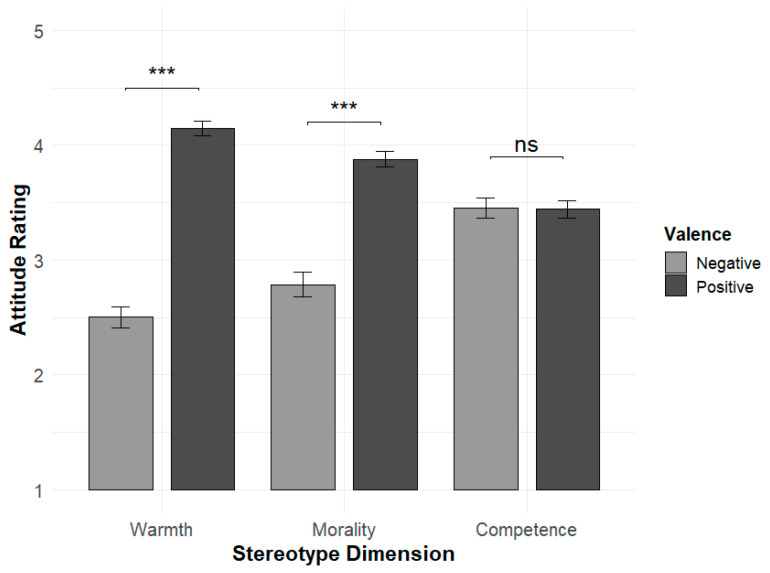
Mean explicit stereotype ratings (±SE) across three stereotype dimensions (Warmth, Morality, and Competence) as a function of attitude valence (Positive vs. Negative). Significant differences between valence conditions were observed for Warmth and Morality, but not for Competence. Error bars represent standard errors of the mean. Asterisks denote significance levels (*p* < 0.001 for ***), and ns indicates non-significance.

Together, these findings support the conceptual distance hypothesis, suggesting that the influence of attitudes on stereotype content is stronger for warmth and morality than for competence (see Figure 3).

**Figure 4:**
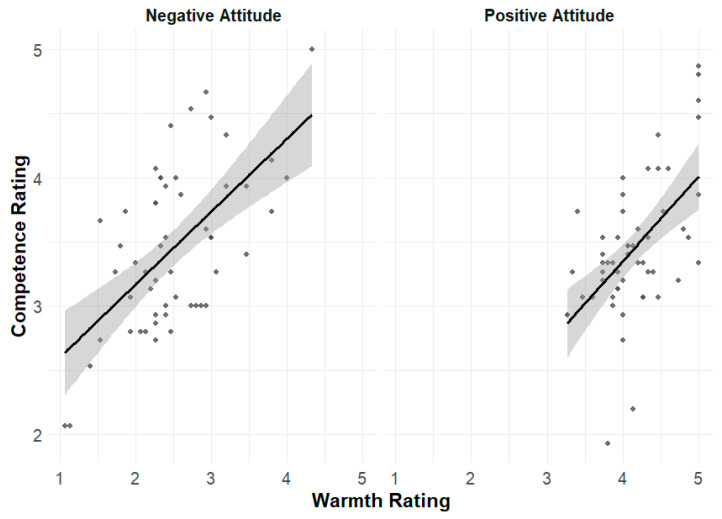
Correlation between warmth and competence stereotype ratings under positive and negative attitude conditions. Each dot represents a participant. Shaded areas represent 95% confidence intervals.

Further, warmth and competence stereotypes were positively correlated under both positive (r = 0.566, *p* < 0.001) and negative (r = 0.608, *p* < 0.001; see Figure 4) conditions, supporting stereotype content model (SCM) predictions.

## 4. Experiment 2

Building on Experiment 1, which investigated whether induced attitudes could causally shape stereotype judgments, Experiment 2 was designed to examine the reverse direction of influence—whether exposure to stereotype content can shape evaluative attitudes. Specifically, this set of within-subject experiments (2a–2c) tested whether trait information related to three core stereotype dimensions—warmth, morality, and competence—could influence participants’ explicit attitudes toward fictional social groups.

### 4.1. Participants

Experiment 2a (warmth dimension) recruited 21 participants (22.03 ± 1.56 years; 11 females), Experiment 2b (morality dimension) recruited 22 participants (21.33 ± 1.68 years; 12 females), of whom 20 were included in the analysis, and Experiment 2c (competence dimension) included 19 students (21.37 ± 1.94 years; 11 females).

### 4.2. Experimental Design and Procedures

Experiments 2a–2c shared an identical procedure, which largely followed that of Experiment 1. The key difference was that participants were first exposed to stereotype trait information of varying valence (positive vs. negative) from one target dimension (warmth, morality, or competence), rather than undergoing attitude conditioning.

After the learning phase, participants rated their overall attitudes toward the fictional group. Each sub-experiment focused on a single stereotype dimension, and the order of conditions was counterbalanced across participants.

### 4.3. Results

To examine whether exposure to stereotype-based trait information influences evaluative attitudes, we conducted a mixed-design ANOVA with trait valence (positive vs. negative) as a within-subjects factor and stereotype dimension (warmth, morality, competence) as a between-subjects factor. The analysis revealed the interaction between valence and dimension was significant, F(2, 57) = 4.40, *p* = 0.017, η^2^ = 0.11, suggesting that the magnitude of the valence effect varied as a function of stereotype dimension. There was a significant main effect of valence, F(1, 57) = 65.80, *p* < 0.001, η^2^ = 0.48, indicating that participants generally reported more favorable attitudes toward social groups described with positive traits compared to those described with negative traits.

**Figure 5:**
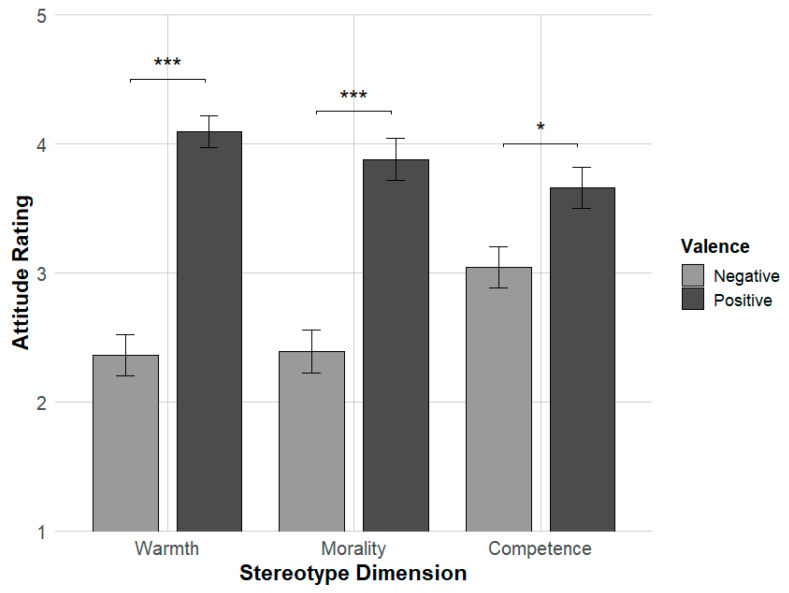
Effects of trait valence (positive vs. negative) on participants’ attitudes across three stereotype dimensions (warmth, morality, and competence). Bars represent mean attitude ratings toward target groups associated with positive or negative trait primes. Error bars indicate ±1 standard error of the mean. Asterisks denote significance levels (*p* < 0.05 for *, and *p* < 0.001 for ***).

To further investigate the interaction, follow-up paired-sample *t*-tests were conducted separately within each dimension. These analyses revealed significant valence effects across all three conditions: attitudes were more positive for groups described with positive traits than with negative traits. Specifically, in the competence condition, the difference was modest but significant, t(18) = −2.35, *p* = 0.030; in the morality condition, the effect was larger, t(19) = −4.94, *p* < 0.001; and in the warmth condition, the strongest valence-based shift in attitudes was observed, t(20) = −6.53, *p* < 0.001 (see Figure 5).

These results provide evidence that stereotype content can shape attitudes under experimental conditions, and importantly, that this effect is not uniform across dimensions. The stronger valence effects observed in the warmth and morality conditions relative to competence support the conceptual distance hypothesis, which posits that evaluative responses are more tightly coupled with warmth and morality than with competence-based information.

## 5. Experiment 3

This experiment aimed to investigate whether evaluative attitudes could causally influence implicit stereotype activation across the dimensions of warmth, morality, and competence, using between-subject design.

### 5.1. Participants

Experiment 3 initially recruited 60 participants. The final sample after outlier removal consisted of 26 participants (23.6 ± 1.68 years, 14 females) in the EC condition and 27 in the control condition (22.4 ± 1.53 years, 16 females). All participants were undergraduate students from South China Normal University, had normal or corrected-to-normal vision, and provided informed consent before participation.

### 5.2. Experimental Design and Procedures

Participants were randomly assigned to an EC or control condition. In the EC condition, group names (e.g., Planet M vs. Planet N) were paired with valenced images. In the control condition, name–name and image–image pairings were used, matching exposure frequency without evaluative content.

Following the learning phase, participants completed stereotype IATs assessing implicit associations between the fictional groups and traits in the three stereotype dimensions: warmth, morality, and competence.

### 5.3. Results

Implicit stereotype activation was assessed using stereotype IATs targeting the dimensions of warmth, morality, and competence. Higher IAT D-scores indicate stronger associations between the positively evaluated group and positive traits, as well as between the negatively evaluated group and negative traits. Thus, higher scores reflect a stronger alignment between evaluative attitudes and stereotype valence. To account for the within-subject structure of the data, a mixed-design ANOVA was conducted with condition (EC vs. Control) as a between-subject factor and dimension (warmth, morality, competence) as a within-subject factor.

The analysis revealed there is no significant interaction effect between condition and dimension. The main effect of condition is significant, F(1, 51) = 16.33, *p* < 0.001, η^2^ = 0.123, indicating that participants in the EC condition exhibited overall stronger stereotype IAT scores than those in the control condition. There was also a significant main effect of dimension, F(2, 102) = 3.42, *p* = 0.038, η^2^ = 0.036, suggesting modest differences in stereotype strength across dimensions.

**Figure 6:**
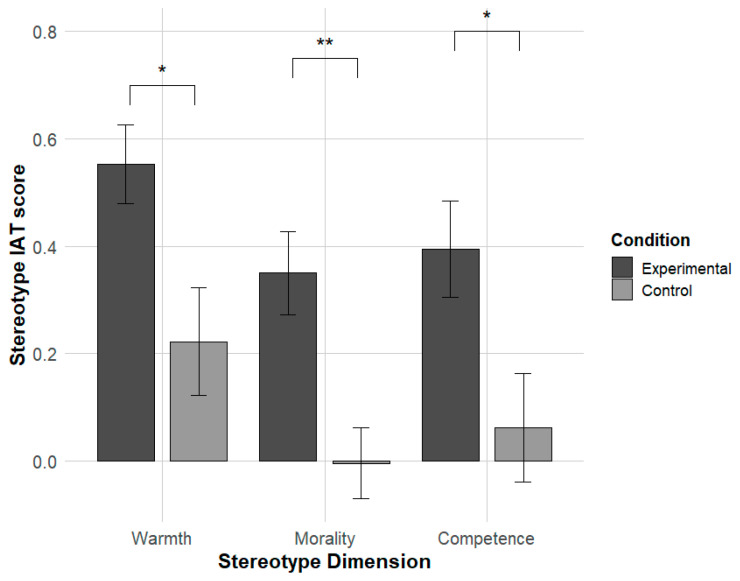
Effect of attitude priming on the induction of implicit stereotypes (warmth, competence and morality). Asterisks denote significance levels (*p* < 0.05 for * and *p* < 0.01 for **).

Post hoc analysis (see Figure 6) revealed that participants in the Experimental condition showed significantly higher IAT D-scores than those in the control condition for warmth (t(51) = 2.65, *p* = 0.010, d = 0.72), morality (t(51) = 3.49, *p* = 0.001, d = 0.96), and competence (t(51) = 2.46, *p* = 0.02, d = 0.67). Together, these findings indicate that induced evaluative attitudes causally enhanced the automatic activation of stereotype content across all three dimensions. Additionally, although the interaction effect was not statistically significant, effect size estimates suggested relatively stronger evaluative influences on warmth and morality than on competence, a pattern consistent with the conceptual distance hypothesis.

**Figure 7:**
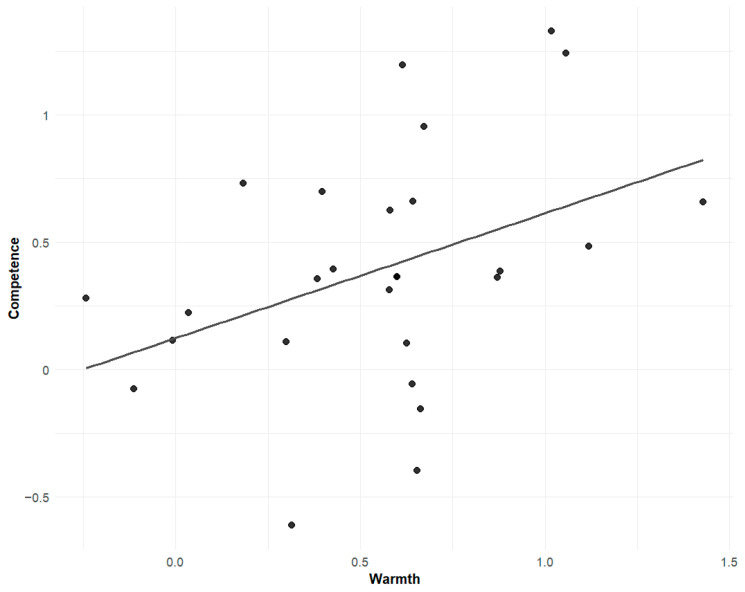
Positive correlation between warmth and competence stereotypes based on implicit measures.

A positive correlation was found between implicit warmth and competence stereotypes (r = 0.40, *p* = 0.037 < 0.05, see Figure 7), consistent with explicit findings in Experiment 1.

## 6. Experiment 4

Experiment 4 aimed to test whether stereotype priming in different dimensions—warmth, morality, and competence—could influence participants’ implicit attitudes. While Experiment 3 demonstrated that evaluative attitudes could activate implicit stereotypes, this experiment examined the reverse direction of causality using IATs as outcome measures. Each of the three sub-experiments (4a–4c) focused on one stereotype dimension to assess whether evaluative meaning could be induced implicitly via stereotype content, using between-subject design.

### 6.1. Participants

Experiments 4a, 4b, 4c initially recruited 60 participants. In experiment 4a, the final sample after outlier removal consisted of 28 participants (22.56 ± 1.63 years, 16 females) in the EC condition and 28 in the control condition (22.90 ± 1.57 years, 17 females). In experiment 4b, the final sample after outlier removal consisted of 26 participants (21.53 ± 1.96 years, 16 females) in the EC condition and 27 in the control condition (22.43 ± 1.58 years, 17 females). In experiment 4c, the final sample after outlier removal consisted of 27 participants (22.29 ± 1.49 years, 17 females) in the EC condition and 26 in the control condition (22.67 ± 1.74 years, 16 females). All participants were undergraduate students from South China Normal University, had normal or corrected-to-normal vision, and provided informed consent before participation.

### 6.2. Experiment Design and Procedures

Experiments 4a–4c were three independent between-subject studies, each involving different participants, designed to test whether stereotype priming in specific dimensions (warmth, morality, and competence) could influence implicit attitudes. The procedure closely followed that of Experiment 3, with the direction of manipulation reversed. Following the learning phase, all participants completed an Attitude IAT to assess implicit evaluations of the fictional groups.

### 6.3. Results

Higher IAT D-scores reflected stronger associations between positively stereotyped groups and positive evaluations, as well as between negatively stereotyped groups and negative evaluations—that is, greater alignment between stereotype valence and implicit attitudes.

We conducted a two-way between-subjects ANOVA on the IAT D-scores, with Condition (experimental vs. control) and Dimension (warmth, morality, competence) as between-subjects factors.

Although no significant main or interaction effects emerged from the omnibus ANOVA, we subsequently conducted exploratory, dimension-specific comparisons to assess whether stereotype priming exerted differential causal effects on implicit evaluations across dimensions. This analytic approach was both theoretically and statistically justified.

**Figure 8:**
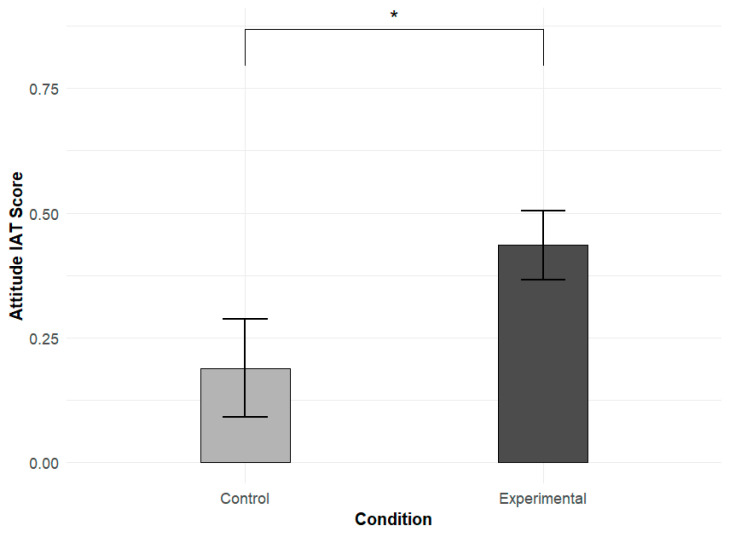
Effect of morality stereotype priming on implicit attitudes. Asterisks denote significance levels (*p* < 0.05 for *).

Theoretically, our predictions were informed by the proposed conceptual distance effect. Specifically, warmth and morality are conceptually more proximal to attitude evaluation, while competence is more distal. Based on this framework, we expected stronger causal effects of stereotype priming on implicit attitudes in the warmth and morality dimensions, justifying separate comparisons within each dimension. Statistically, the experimental design assigned independent groups of participants to each stereotype dimension, with no within-subject factors involved. Therefore, it was appropriate to conduct independent-samples *t*-tests within each dimension to directly compare implicit evaluations between the experimental and control conditions. The analysis revealed a significant difference in the morality dimension between the experimental and control groups (t(51) = 2.03, *p* = 0.047, d = 0.56) (see Figure 8). No significant differences were observed in the warmth or competence dimensions.

Together, Experiment 3 and Experiment 4 revealed that while implicit attitudes influence implicit stereotypes, only morality stereotypes could shift implicit attitudes. These patterns partially support the conceptual distance effect, though less robustly than in explicit measures.

## 7. Discussion

Previous studies have reported mixed findings regarding the relationship between attitudes and stereotypes, with some suggesting no association and others providing evidence of a strong correlation or even causality (Kurdi et al., 2019; Phills et al., 2020). Unlike prior work that typically examined general evaluative valence, our study investigated this relationship from the perspective of specific stereotype dimensions—morality, warmth, and competence—using both explicit and implicit measures. By employing fictional groups and evaluative conditioning, we minimized the influence of preexisting associations, allowing participants to form attitudes or stereotypes in a controlled environment. Across two studies, we found bidirectional causal effects under experimental manipulation between attitudes and stereotypes using explicit measures and a partial relationship using implicit measures, with the morality dimension showing the most robust effect.

These findings support the conceptual distance effect, which proposes that semantic proximity between constructs determines the strength of their causal connection. Our pre-experimental assessments revealed that morality and warmth were conceptually closer to attitudes than competence, a pattern that was reflected in the results. In Experiment 1 and Experiment 2, explicit measures showed bidirectional influence patterns across all dimensions, but the effects were stronger for morality and warmth. In Experiment 3 and Experiment 4, although attitudes influenced implicit stereotypes in all three dimensions, only moral stereotypes influenced implicit attitudes. This asymmetry is consistent with the spreading activation model and Greenwald’s Unified Theory, which posits that closely connected nodes in semantic memory are more likely to activate one another, resulting in stronger influence pathways (Greenwald et al., 2002).

The weaker effects observed in implicit stereotype-to-attitude conditions may reflect both cognitive and methodological factors. According to dual-process models, explicit reports do not always align with implicit cognitions (Gawronski & Bodenhausen, 2011). Additionally, individual differences in attitude strength and the limitations of the implicit association test (IAT) may have contributed to reduced sensitivity in detecting effects across dimensions. Previous research has shown low correlation between different implicit measures (Schimmack, 2021), suggesting that future studies should explore a broader range of tools, such as affective priming or other indirect methods, to better capture the nuances of stereotype–attitude dynamics. While our current framework draws on the classic spreading activation model to explain how conceptual proximity shapes causal linkages, we acknowledge that it offers a simplified account of semantic structure. Contemporary advances in distributional semantics—such as word embeddings (e.g., Word2Vec, GloVe, BERT)—provide more precise, data-driven methods for quantifying semantic similarity (Charlesworth & Banaji, 2022; Qin & Tam, 2025). Our notion of conceptual distance is compatible with these approaches, and future research could leverage such models to refine both the measurement and theoretical grounding of stereotype–attitude dynamics. This integration would help bridge classic associative theories with recent developments in computational semantics.

Interestingly, our exploratory analysis revealed a stable positive correlation between warmth and competence evaluations in both explicit and implicit conditions. This finding is consistent with Judd et al. (2005), who reported similar patterns in evaluations of individual targets, though past evidence has been mixed. Importantly, we interpret this correlation as reflecting an association between stereotype dimensions themselves, rather than a direct indicator of causal influence between attitudes and stereotypes. Therefore, this result does not conflict with the conceptual distance framework that informs our core analyses.

### Limitations and Future Directions

Despite the strengths of our design, several limitations should be noted. Conceptual distance reflects evaluative proximity between traits and valence within a specific cultural context. We acknowledge that such distances may vary across societies depending on social norms, values, and language use. Our findings should therefore be interpreted as context-bound rather than universally fixed. Importantly, this does not undermine the conceptual distance effect but suggests that it may take different forms in different cultural settings. Future research should examine whether the observed pattern—where morality and warmth are evaluatively closer to attitudes than competence—replicates across diverse cultural contexts. The EC paradigm, though widely supported, may yield variable effects depending on the stimuli and context, and some stereotype traits—particularly within the competence dimension—may not have fully captured the intended meaning. Additionally, while the inclusion of morality as a third dimension beyond the classical SCM framework produced novel insights, future research could refine stereotype dimensionality further, especially across different cultural contexts. Moreover, although the use of fictional groups allowed us to avoid preexisting biases and ensure experimental control, it may limit the external validity of our findings, particularly in real-world intergroup contexts. This trade-off should be addressed in future replications with more ecologically valid targets. Finally, the present findings offer promising avenues for real-world applications in domains such as education, diversity training, and public policy. Given that stereotype dimensions such as morality and warmth are more evaluatively aligned with attitudes, interventions can strategically focus on these dimensions to trigger broader cognitive changes. For example, promoting moral exemplars or enhancing perceived warmth in marginalized groups may lead to spontaneous attitude shifts, even without directly targeting evaluation. Additionally, the demonstrated asymmetry in implicit processes highlights the need for refined intervention tools that are sensitive to how conceptual proximity shapes automatic bias. These insights provide a theoretical foundation for designing cost-effective, scalable programs aimed at prejudice reduction in schools, organizations, and beyond.

## 8. Conclusions

The present study investigated the bidirectional causal effects under experimental manipulation between attitudes and stereotype dimensions, and interpreted the divergent patterns through the lens of the “conceptual distance” framework. The findings demonstrated stable bidirectional influence patterns between attitudes and stereotypes, with variation in the strength of experimentally induced influence across stereotype dimensions aligning with the conceptual distance effect. These results offer both theoretical insight and practical guidance, highlighting the importance of considering conceptual proximity in future research on social cognition and prejudice reduction.

At the same time, these conclusions should be interpreted with appropriate caution. As with all experimental research, unmeasured confounding variables and broader cultural contexts may influence both attitudes and stereotypes, thereby limiting the external validity of our findings. The conceptual distance framework, as proposed here, should therefore be viewed as a preliminary theoretical tool for understanding attitude–stereotype dynamics—one that calls for further empirical validation across diverse cultural settings and methodological approaches. Future research is encouraged to explore how conceptual proximity operates in real-world intergroup contexts and whether similar patterns emerge beyond the experimental paradigm employed here.

## Data Availability

The data that support the findings of this study are available from the corresponding author upon reasonable request.

## Supplementary Materials

The following supporting information can be downloaded at: https://www.mdpi.com/article/10.3390/bs16020287/s1, Table S1: trait words of each stereotype dimension; Table S2: Attitude evaluation items.

## Abbreviations

The following abbreviations are used in this manuscript:

SCM	Stereotype Content Model
LAPS	International Affective Picture System
EC	Evaluative Conditioning
IAT	Implicit Association Tests
