# Supplementary material for: The ‘Conceptual Distance Effect’ in the Causal Effects Under Experimental Manipulation Between Attitude and Stereotype
Source: Behav Sci (Basel). 2026 Feb 17;16(2):287. doi: 10.3390/bs16020287 (PMC12938441; doi:10.3390/bs16020287)
Supplement: Supplementary file 1 [file behavsci-16-00287-s001.zip › behavsci-4065624-supplementary.pdf]

Table S1. trait words of each stereople dimension

能力			温情			道德		
正性词	分数	负性词	正性词	分数	负性词	正性词	分数	负性词
效率高	4.37	效率低	热情的	4.8	漠然的	正义的	4.68	邪恶的
熟练的	4.27	笨拙的	热心的	4.68	冷血的	诚实的	4.61	虚伪的
悟性强	4.24	悟性弱	友好的	4.17	敌对的	公正的	4.56	偏私的
能干的	4.24	无能的	诚挚的	4.05	虚假的	廉正的	4.44	腐败的
外语好	4.15	外语差	温暖的	4.05	冷漠的	高尚的	4.39	下流的
行动快	4.1	行动慢	支持的	4	反感的	贤德的	4.37	奸佞的
适应快	4.1	适应慢	亲和的	3.85	凶恶的	正直的	4.37	奸邪的
有才干	4.07	平庸的	大方的	3.8	小气的	忠诚的	4.32	狡诈的
博学的	4.02	无知的	真诚的	3.8	虚伪的	无私的	4.2	自私的
见识广	3.93	没见识	慷慨的	3.61	吝啬的	孝敬的	4.12	忤逆的
有头脑	3.88	没头脑	温厚的	3.41	苛刻的	善良的	4.12	恶毒的
天才的	3.83	庸才的	有礼貌	3.29	无礼的	仁爱的	4.12	暴虐的
有主见	3.71	没主见	耐心的	3.29	易怒的	高洁的	4.12	下贱的
聪明的	3.68	愚蠢的	宽容的	3.17	严厉的	磊落的	4.1	阴暗的
机敏的	3.68	迟钝的	脾气好	3.05	脾气差	仁厚的	3.95	苛刻的

Table S2. Attitude evaluation items

positive attitude evaluation
请仔细阅读以下各个题目，根据您的真实情况对 X 星球的人进行打分，您的资料将只用于科学研究。所有备选答案均为“非常不符合、比较不符合、介于符合于不符合之间、比较符合、非常符合”。“非常不符合”为 1，“非常符合”为 5，请根据实际情况在每个题目后填写 1-5。
认知：
我觉得他们应该是热爱生活的
我觉得他们身边应该有很多朋友。
我觉得他们遇到困难常常敢于应对。
我觉得他们在生活中阳光开朗，受人欢迎。
我觉得他们是情绪稳定的人。

我觉得他们爱好广泛，快乐有趣。
我觉得他们对自己很有信心，不会轻易否定自己。
情感：
我很喜欢这样的人。
如果我有这样的朋友或者同学我会很开心。
我会被他们带动，不由自主地向他们靠近。
我相信他们能很好地处理人际关系。
我相信他们总能给我带来快乐和安慰。
我非常乐意在生活中和这样的人交朋友。
我喜欢和他们一起体验和探索新鲜事物。
行为：
我想主动地了解他们。
他们有困难向我求助我会十分乐意提供帮助。
我会主动和这样的人交朋友。
我会选择这样的人作为工作或者学习的伙伴。
我会真诚热情地对待他们。
我会包容他们的一些缺点和错误。
我愿意和他们一起面对困难和挑战。
negative attitude evaluation
请仔细阅读以下各个题目，根据您的真实情况对 L 星球的人进行打分，您的资料将只用于科学研究。所有备选答案均为“非常不符合、比较不符合、介于符合于不符合之间、比较符合、非常符合”。“非常不符合”为 1，“非常符合”为 5，请根据实际情况在每个题目后填写 1-5。
认知：
我觉得他们对生活缺乏热爱。
我觉得他们是不友好的，朋友可能很少。
我觉得他们可能常常消极地看待事情。
我觉得他们在生活中偏执、狭隘，不好接近。
我觉得他们不是擅长控制情绪的人
我觉得他们在生活中可能没有什么爱好，乏味无趣。
我觉得他们的内心深处是自卑的，与表面表现的相反。
情感：
我不喜欢这样的人。
如果我发现身边有这样的人我的心情会受到影响。

生活中我可能会有意远离他们。
我对这样的人的人际交往能力持怀疑或者否定态度。
我深信他们带给我的更多会是不好的影响。
我不愿意在生活中和这样的人交朋友。
我不希望和这样的人共事。
行为：
我可能不会主动地了解他们。
看到他们遇到困难我不会很情愿地提供帮助。
我不会主动和这样的人交朋友。
我不会选择这样的人作为工作或者学习的伙伴。
我不会像对待其他人那样热情地对待他们。
我对他们的缺点和错误相对来说可能会缺少耐心和包容。
我不愿意和他们一起应对困难和挑战。
